# Comparative safety and efficacy of manual therapy interventions for cervicogenic headache: a systematic review and network meta-analysis

**DOI:** 10.3389/fneur.2025.1566764

**Published:** 2025-05-16

**Authors:** Xueliang Xu, Yan Ling

**Affiliations:** ^1^Department of Rehabilitation III, Hospital of Chengdu University of Traditional Chinese Medicine, Chengdu, China; ^2^Department of Pediatrics, Hospital of Chengdu University of Traditional Chinese Medicine, Chengdu, China

**Keywords:** cervicogenic headache, Neck Disability Index, Flexion-Rotation Test, manual therapy, meta-analysis

## Abstract

**Objective:**

To evaluate and compare the safety and efficacy of spinal manipulation, mobilization, and massage for the management of cervicogenic headache (CGH) using meta-analytic techniques.

**Methods:**

Comprehensive searches were conducted in Cochrane, Embase, PubMed, and ClinicalTrials.gov to identify studies investigating the effects of manipulation, mobilization, and massage on pain, disability, and physical function in patients with CGH. Key outcomes included pain severity (visual analog scale, VAS), Neck Disability Index (NDI), Flexion-Rotation Test (FRT), and Headache Disability Inventory (HDI) at various follow-up timepoints.

**Results:**

Fourteen studies totaling 1,297 CGH patients were included. Standard pairwise meta-analysis revealed that sustained natural apophyseal glides (SNAG) mobilization produced significantly greater improvements compared to non-SNAG interventions in VAS (MD = 1.73, 95%CI: 1.05, 2.40), NDI (MD = 8.55, 95%CI: 2.73, 14.37), FRT (MD = −7.22, 95%CI: −9.38, −5.07), and HDI (MD = 9.29, 95%CI: 3.64, 14.95), with benefits maintained over time. Network meta-analysis showed that for VAS improvement, the surface under the cumulative ranking curve (SUCRA) probabilities were: cervical spine manipulation (CSM, 98.9%), mobilization (67.3%), exercise (21.0%), and massage (12.8%). For NDI, the SUCRA scores were: CSM (82.2%), mobilization (57.2%), exercise (6.7%), and massage (53.9%). CSM exhibited significantly greater VAS reductions compared to exercise, massage, and mobilization, while mobilization was superior to exercise and massage for VAS. For NDI, CSM was significantly better than exercise, but no other between-group differences were observed.

**Conclusion:**

In patients with CGH, SNAG mobilization can significantly improve pain and function, with benefits maintained in the long-term. Additionally, CSM may be the most effective short-term intervention for reducing pain and disability compared to mobilization, massage, and exercise, although clinician expertise appears to be an important factor.

**Systematic Review Registration:**

DOI: 10.37766/inplasy2025.3.0079.

## 1 Introduction

Cervicogenic Headache (CGH) denotes a type of headache syndrome characterized by the transmission of pain sensations from the cervical region to the head (1). Typically arising from issues such as cervical muscle tension, cervical facet joint osteoarthritis, cervical disc protrusion, and other cervical pathologies, CGH commonly manifests alongside symptoms like neck pain, restricted cervical mobility, and cervical-thoracic stiffness (2). These symptoms tend to emerge following sustained neck postures, repetitive cervical movements, or physical exertion, often accompanied by limitations in cervical range of motion. CGH presents as a non-throbbing, unilateral headache originating from the cervical spine and extending to the occipital, temporal, and periorbital regions, representing a predominant headache subtype. The precise pathophysiological mechanisms underlying cervicogenic headache remain elusive but are intricately linked to pathological changes within cervical anatomical structures, encompassing factors such as cervical facet joint dysfunction, myofascial inflammatory or mechanical compression of cervical nerves, and musculoskeletal impairments (3).

Primary management strategies for CGH include physical therapy interventions, comprising manual techniques and therapeutic exercises, which have demonstrated efficacy in alleviating cervicogenic headaches (4). Moderate-quality evidence supports the use of manual therapies (e.g., cervical muscle relaxation techniques, spinal manipulation), targeted exercise modalities (static and dynamic stretching exercises for cervical and scapular joints or upper extremities), and low-load endurance exercise programs to ameliorate headache intensity, pain, disability, as well as frequency and duration (5).

The foundational principles of manual therapy encompass Manipulation, Mobilization, Massage, among other modalities, each distinct yet interconnected in their approaches (6). Manipulation involves high-velocity, low-amplitude maneuvers, whereas Mobilization entails low-velocity, high-amplitude movements. Massage techniques employ varied pressure applications through kneading, stroking, percussion, or other methods to elicit desired muscular responses (7). Owing to diverse anatomical and physiological dysfunctions, manual therapy emerges as a prevalent treatment modality for CGH patients, despite conflicting evidence concerning its effectiveness. “Sustained Natural Apophyseal Glides” (SNAGs), a Mobilization-based therapeutic technique pioneered by Mulligan, posits that subtle joint misalignments following neck trauma or strain contribute to restricted motion and pain, imperceptible through conventional assessments (8). Mulligan believes that they are not visible through palpation or X-rays. However, with the correct joint mobilization, pain-free functional activity can be achieved, and with repeated application, sustained improvement is possible (9). Distinguished from conventional joint mobilizations solely targeting joint structures, Mulligan’s approach integrates joint mobilization with soft tissue release, effectuated by applying pressure to spinal processes in a weight-bearing position (10), inducing synchronous sliding movements in minor joints as part of the SNAGs protocol (11). Meta-analytical findings corroborate the efficacy of SNAGs in alleviating pain, reducing disability, and enhancing quality of life in CGH patients (12), notwithstanding the inconclusive evidence regarding differential efficacy among distinct manual therapy modalities (13).

## 2 Methods

This study is a meta-analysis of randomized controlled trials (RCTs) conducted in accordance with the Preferred Reporting Items for Systematic Reviews and Meta-Analyses (PRISMA) guidelines.

### 2.1 Search strategy

A literature search was conducted to identify studies on the effects of Manipulation, Mobilization, and Massage in alleviating the frequency, severity, and functionality of patients with Cervicogenic Headaches (CEHs) in Cochrane, Embase, PubMed, and Clinicaltrials.gov. These databases were searched for studies assessing the effects of manipulation, mobilization, and massage on CGH.

The following search terms were used: ((Cervicogenic headaches) OR (Cervicogenic headache)) AND (((((Manipulation) OR (Mobilization)) OR (Massage)) OR (SNAG))). The search deadline was July 1, 2024. The review included studies without any language restrictions, ensuring a more comprehensive search and inclusion of international studies.

### 2.2 Study selection criteria

Literature inclusion was determined based on the PICOS criteria. P (Patients): studies included had to focus on patients diagnosed with CGH according to accepted diagnostic criteria. This ensures the studies are highly relevant to the topic of interest; I (Intervention): the intervention must involve one or more manual therapies such as manipulation, mobilization, or massage. The use of SNAGs was also included; C (Control): The control could include other manual therapies, sham treatments, exercise interventions, or no intervention. This broad control category allowed for comparison across different interventions. O (Outcome): the study outcomes needed to include pain and functional measures post-intervention, specifically using tools such as the Neck Disability Index (NDI), Headache Disability Inventory (HDI), Numerical Pain Rating Scale (NPRS), and the Flexion-Rotation Test (FRT); S (Study design): Only randomized controlled trials (RCTs) were included to ensure the highest level of evidence. RCTs are considered the gold standard in clinical research for assessing the effectiveness of interventions.

Studies were excluded for the following reasons: (1) headaches diagnosed as migraines or tension-type headaches from other causes, (2) case reports or studies with fewer than 5 participants, (3) studies not published in peer-reviewed journals, and (4) studies not published in English.

Impact of control group variability on comparison: inconsistent sham interventions: different control group settings across studies (e.g., placebo mobilizations vs. minimal interventions) introduce variability, making it hard to determine if observed effects are from the manual therapy (like snag) or the control treatments themselves. Sham groups and placebo effect: if some control groups receive more active treatments (e.g., mild mobilization), it could mask the effects of the manual therapy. This makes it difficult to know if improvements are due to the experimental therapy or the control intervention being unexpectedly effective. Bias in interpretation: the variation in control conditions can lead to biased conclusions. If one study uses a minimal control while another uses a more active placebo, it may make the experimental treatment seem more effective than it actually is.

To improve the validity and comparability of future research on SNAG therapy for CGH, studies should adopt standardized sham interventions (e.g., “non-specific mobilization”) to ensure consistent controls, implement rigorous double-blinding procedures for both participants and assessors to minimize bias, incorporate active comparators like spinal manipulation to better evaluate relative efficacy, and standardize patient cohorts based on clinical characteristics such as headache severity and duration. These methodological refinements would enhance study reliability and facilitate more meaningful comparisons across trials investigating SNAG’s therapeutic effectiveness.

Revised network meta-analysis strategy: subgroup analysis: group studies by control type to see how different controls impact treatment outcomes. This would clarify how snag compares under different conditions. Sensitivity analysis: conduct sensitivity analyses to explore how variations in control conditions affect results. This helps adjust the findings and makes the conclusions more reliable.

### 2.3 Outcome measures

Headache Disability Inventory (HDI): a self-report questionnaire assessing the impact of headaches on daily activities. Higher scores indicate greater disability caused by headaches.

Neck Disability Index (NDI): a self-report questionnaire measuring limitations in daily activities due to neck pain. Higher scores indicate greater disability.

Numerical Pain Rating Scale (NPRS): a scale quantifying pain intensity using a numerical scale, providing a standardized measure of pain severity. Higher scores indicate greater pain intensity.

Flexion-Rotation Test (FRT): used to assess dysfunction in the C1-C2 motion segment, represented by the angle of rotation in neck flexion position, with a normal value around 45° and reduced angles indicating dysfunction (14).

### 2.4 Data extraction and quality assessment

Data were extracted into a predefined Excel sheet, including first author, publication date, patient characteristics, sample size, patient age, interventions, intervention duration, outcome measures, etc. Cross-checking was performed on extracted data, and discrepancies were resolved by a third reviewer.

Two authors independently assessed bias risk using the Cochrane risk of bias tool. Bias risk was evaluated across 7 items under 6 domains: selection (including random sequence generation and allocation concealment), performance (blinding of participants and personnel), detection (blinding of outcome assessment), attrition (completeness of outcome data), reporting (selective reporting of results), and other biases. Each item was judged as “low risk of bias,” “high risk of bias,” or “unclear” based on bias assessment criteria.

### 2.5 Statistical methods

This study compares the efficacy of different manual therapy modalities in CGH. Network meta-analysis is the preferred method, but due to substantial differences in methods and outcomes of sham interventions among studies, there may be considerable heterogeneity that could complicate interpretation and affect conclusions. Therefore, this study is divided into two parts: a conventional meta-analysis comparing the efficacy of SNAGS vs. non-SNAGS, and a network meta-analysis comparing the efficacy of CSM, Mobilization, and Massage.

Conventional meta-analysis was conducted using the R meta package, and figures were generated. Heterogeneity among studies was analyzed using Q and I2 tests. I2 values were interpreted as follows: 0 indicates variation due to sampling error only; <0.25 suggests low heterogeneity; between 0.25 and 0.5 suggests moderate heterogeneity; >0.5 indicates high heterogeneity. Subgroup analyses were conducted for moderate to high heterogeneity, using random-effects models, while fixed-effects models were used for no to low heterogeneity. Funnel plots were used to assess publication bias, with Begg’s test and Egger’s test conducted when including ≥10 studies, and visual inspection for symmetry when fewer than 10 studies. Standardized mean differences (SMD) were used for continuous variables, and risk ratios (RR) for categorical variables. Statistical significance was considered when SMD did not include 0 and RR did not include 1. Stata software was utilized for network meta-analysis, generating network plots, calculating Surface Under The Cumulative Ranking (SUCRA), creating SUCRA plots, and pairwise comparison forest plots. In the network plot, the size of points represents sample sizes, lines indicate direct comparisons, and the thickness of lines represents the number of studies included. Higher SUCRA values indicate better rankings.

## 3 Results

### 3.1 Literature screening process

**Figure 1:**
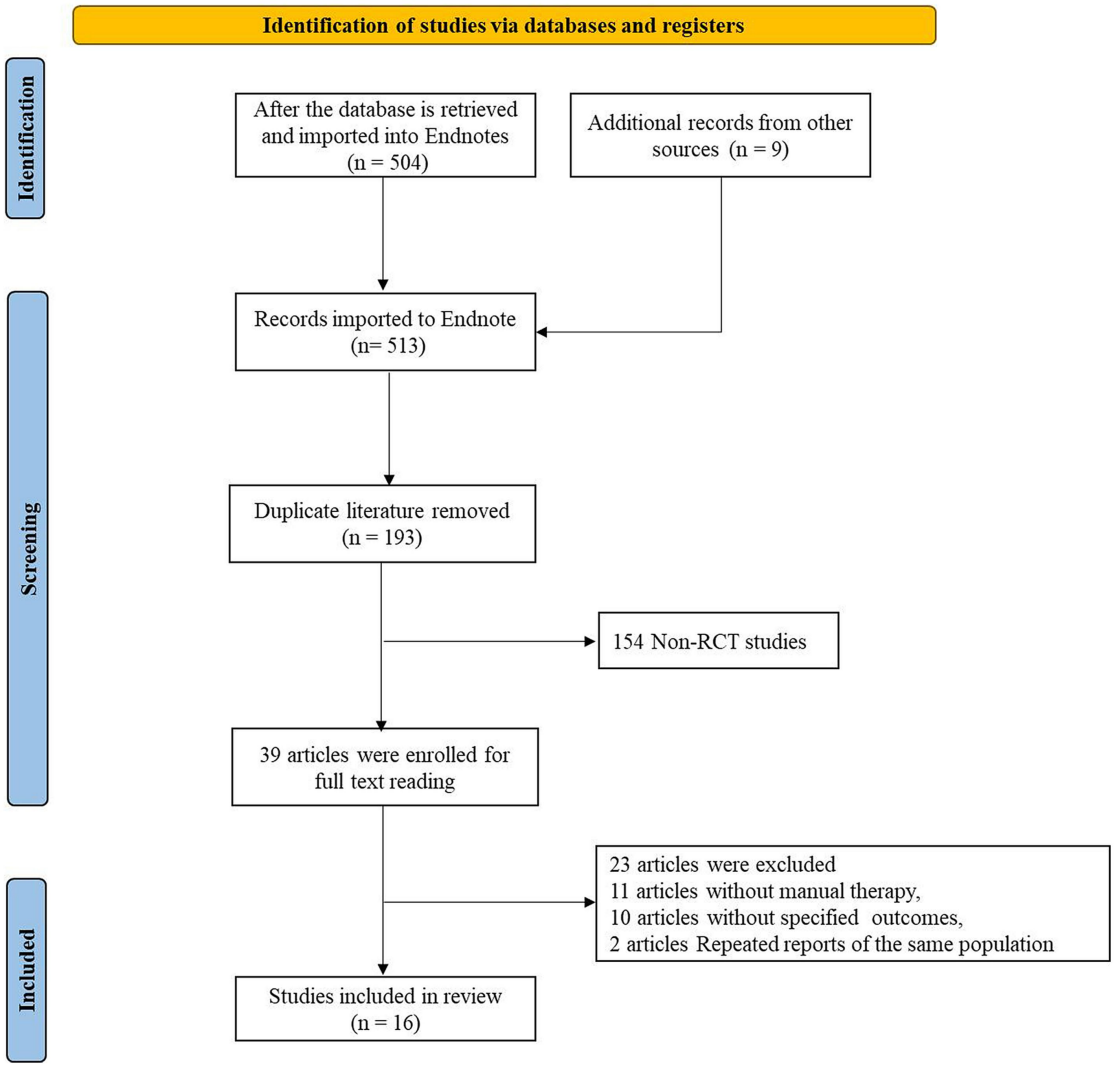
Basic information of included literature chart.

A total of 513 articles were initially retrieved from the databases. After eliminating duplicates using Endnote, 193 articles underwent preliminary screening. Upon reviewing titles and abstracts, 157 articles were excluded for reasons such as not involving clinical studies, lacking manual therapy utilization, or not being RCTs. Following a full-text review of the remaining 36 articles, 22 were further excluded due to various reasons like data extraction challenges, inadequate intervention durations, or data unavailability, resulting in the inclusion of 16 articles. The flowchart illustrating the literature inclusion process is depicted in Figure 1.

### 3.2 Basic information of included literature

**Figure 2:**
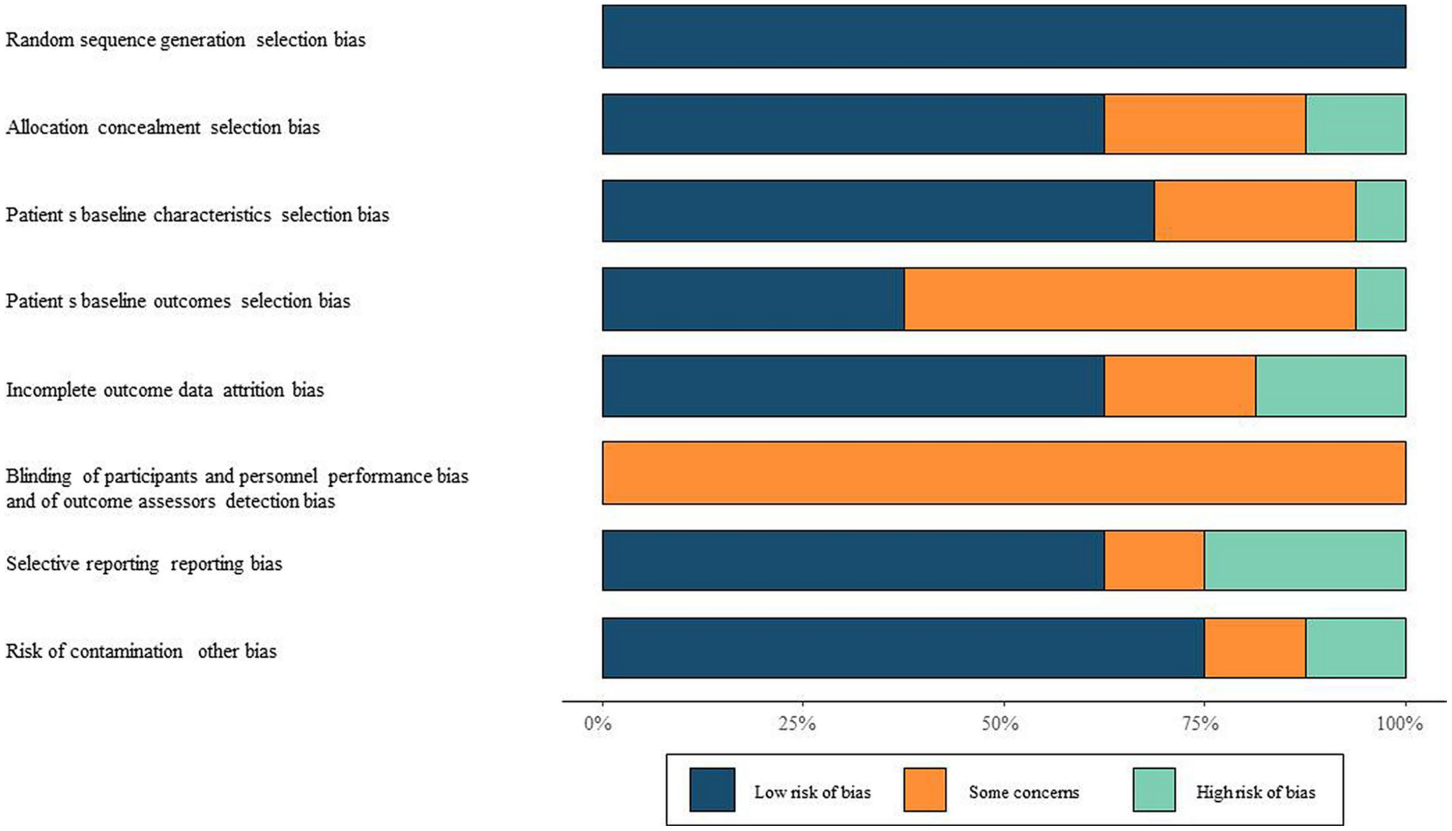
Risk of bias assessment of included literature ROB chart.

Among the 16 included articles, a total of 769 subjects were involved with an average age range spanning from 21.8 to 48.4 years. Manual therapy methods in the included studies encompassed Manipulation, Mobilization, SNAGs, Massage, among others. Table 1 presents the basic information of the included literature. The quality assessment of the included studies using the Risk of Bias (ROB) tool is illustrated in Figure 2.

**Table 1:** Characteristics of included studies.

Author	Year	Intervention	*n*	Age	Duration and frequency
Dunning JR (25)	2016	Manipulation	58	34.1 (12.6)	6–8 sessions over 4 weeks
		Mobilization and exercise	52	36.4 (10.0)	
Kashif M (26)	2022	Mulligan SNAGs	20	22.20 (1.64)	3 times a week for a total of 20 min per performance for 12 times in 4 weeks
		Placebo treatment	20	21.80 (1.54)	
Christian N (27)	2017	Mulligan SNAGs	8	26.8 (1.5)	Treatment for 1 week
		Active neck Range of motion exercises	8	31 (2.09)	
Malo-Urriés M (28)	2017	Translatoric mobilizations	41	42.49 (15.61)	30-min treatment consisting of 30-s series of translatoric mobilizations of the upper cervical spine with 10-s rest periods between sets
		No treatment	41	40.59 (15.10)	
Youssef EF (29)	2013	Passive spinal mobilizations mobilization	18	32.4 (6.5)	Two sessions/week for 6 weeks
		Massage	18	31.0 (3.49)	
Lerner-Lentz A (21)	2021	Mobilization	24	47.5 (17.7)	Two sessions, uncertain timing
		Manipulation	21	48.4 (15.5)	
McDevitt AW (30)	2022	Thoracic spine manipulation	24	34.96 (9.38)	1–2 times weekly for up to 4 weeks
		Hold	24	33.88 (11.79)	
Nambi G (31)	2024	Cervical spine manipulation	32	35.6 (3.8)	6 sessions over 3 weeks
		Conventional physiotherapy	32	36.2 (3.7)	
Satpute K (32)	2024	Mulligan SNAGs	33	40 (11)	6 treatment sessions as per group allocation spread over 4 consecutive weeks
		Exercise	33	40 (11)	
Shin EJ (33)	2014	SNAGs	20	48.20 (7.79)	3times per week, 20 min per performance, a total of 12 times in 4 weeks
		Placebo SNAGs	20	48.05 (6.81)	
Kirthika V (34)	2018	Mulligan SNAGs	12	26.2 (6.8)	3 times per session/day×5 days/week for 4 weeks’ duration
		Muscle energy technique	12	25.7 (7.1)	
Khalil M (35)	2019	Mulligan SNAGs	15	42.53 (7.15)	9 sessions every other day for 4 weeks
		Traditional treatment	15	41.6 (6.62)	
Murtza S (36)	2024	Rocabado	18	40.06 (8.93)	2 treatment sessions per week with a maximum of 16 treatment sessions over 8 weeks
		SNAGs	18	40.39 (10.59)	
Jin X (37)	2023	SNAGs	20	30.05 (6.75)	Once per day, 10 times for one session
		Health promotion	20	27.19 (4.76)	
Rani M (38)	2022	Spinal mobilization	20	41.45 (13.21)	4 weeks, 4 times a week (16 sessions) and each treatment session lasted for approximately 30 min
		SNAGs	20	38.55 (9.28)	
		Motion exercises	20	38.65 (13.89)	
Hall T (39)	2007	Shame SNAGs	16	38 (14)	Daily self-administration for 12 months
		SNAGs	16	33 (11)	

### 3.3 SNAG vs. non-SNAG

Out of the included studies, 10 articles reported the use of SNAG with control groups involving SNAG, exercise, soft tissue massage, Mobilization, etc. Follow-up durations ranged from a minimum of 1 week to a maximum of 12 months. Based on the follow-up time, data were categorized into short-term (≤4 weeks), medium-term (4–24 weeks), and long-term (≥24 months), followed by subgroup analysis.

**Figure 3:**
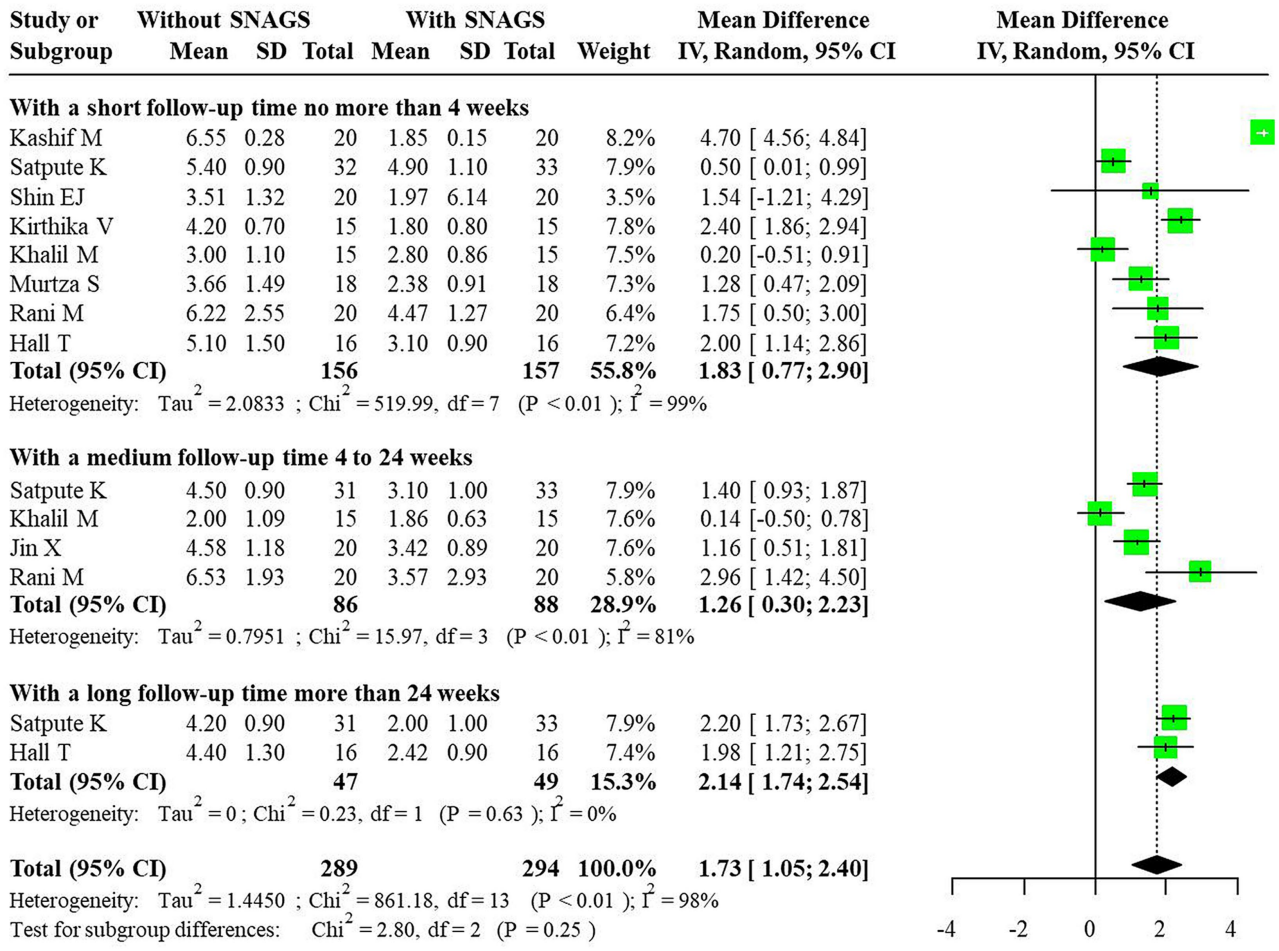
Forest plot of VAS after SNAG and non-SNAG interventions.

For the VAS indicator, significant heterogeneity was detected, necessitating the use of a random-effects model to combine effect sizes. The studies affirmed that SNAGs significantly reduced VAS scores, with an overall combined mean difference (MD) of 1.73 (95% CI: 1.05, 2.40). The MDs for short-term, medium-term, and long-term were 1.83 (95% CI: 0.77, 2.90), 1.26 (95% CI: 0.30, 2.23), and 2.14 (95% CI: 1.74, 2.54), respectively. The relationship between intervention duration and VAS scores post-SNAG and non-SNAG interventions is depicted in Figure 3.

**Figure 4:**
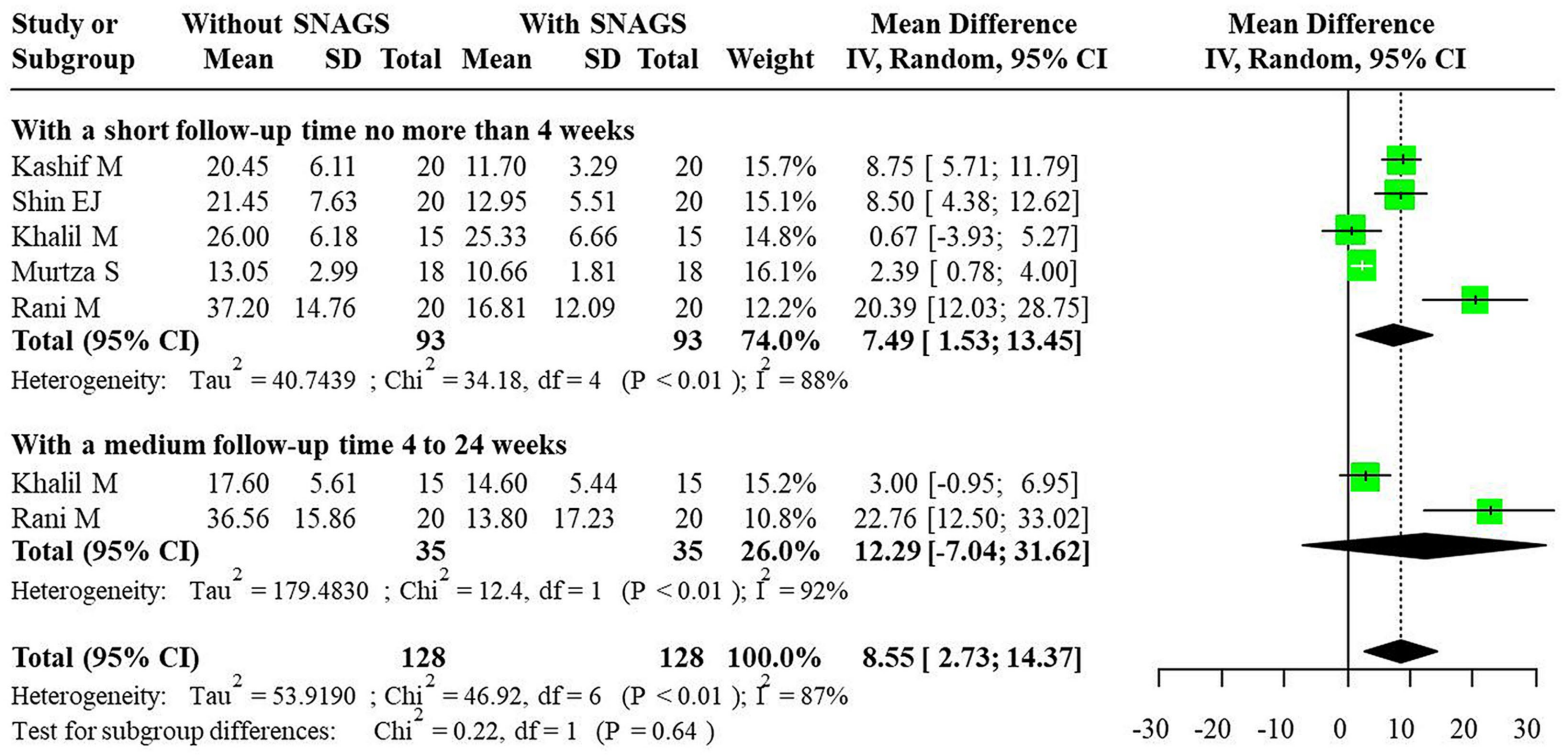
Forest plot of NDI after SNAG and non-SNAG interventions.

For the NDI indicator, significant heterogeneity was also observed, and a random-effects model was employed for combining effect sizes. The studies indicated that SNAGs significantly reduced NDI scores, with an overall combined MD of 8.55 (95% CI: 2.73, 14.37). The MDs for short-term and medium-term were 7.49 (95% CI: 1.53, 13.45) and 12.29 (95% CI: −7.04, 31.62), respectively, with no reports on long-term outcomes. The relationship between intervention duration and NDI scores post-SNAG and non-SNAG interventions is shown in Figure 4.

**Figure 5:**
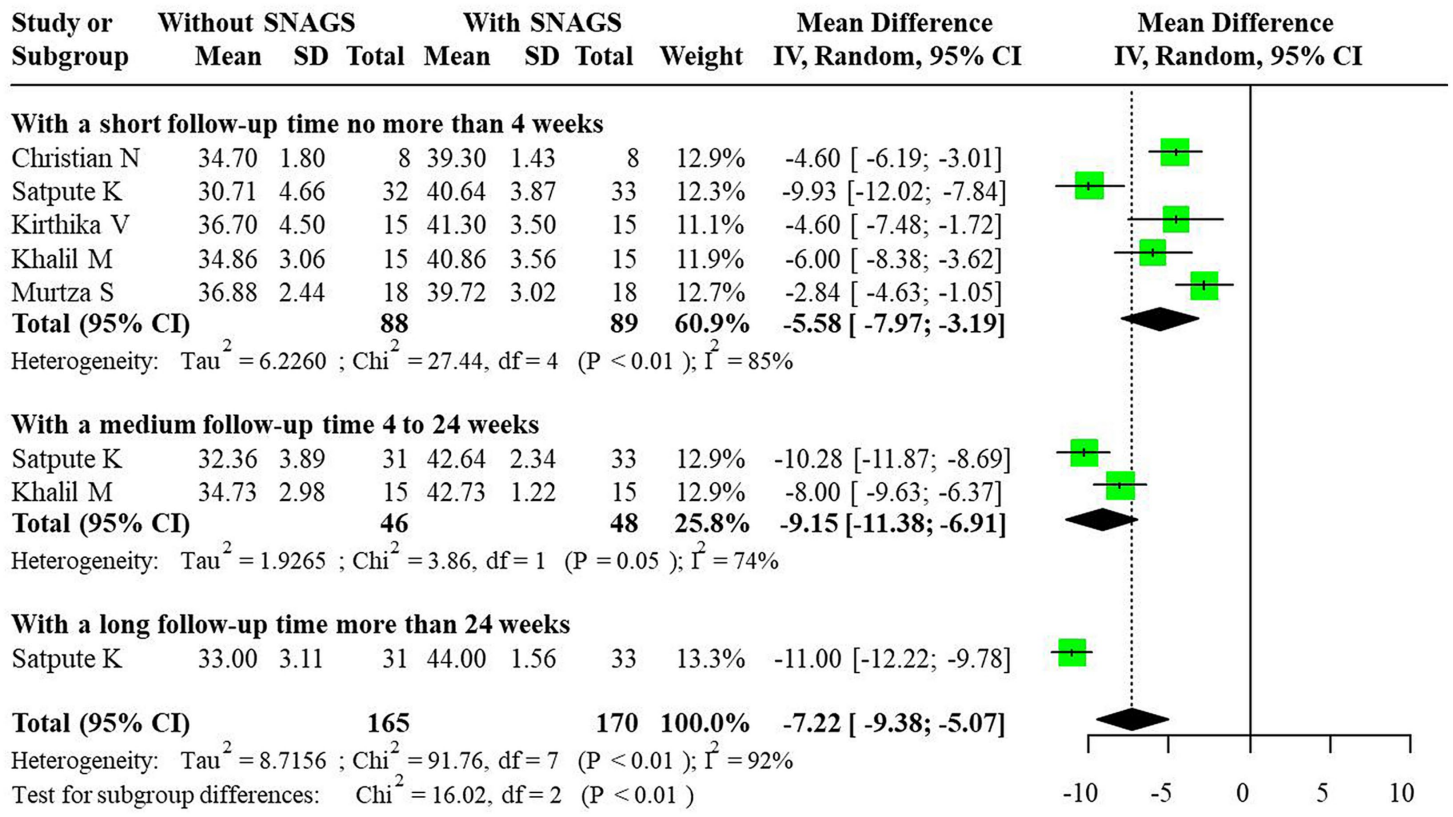
Forest plot of FRT after SNAG and non-SNAG interventions.

For the FRT indicator, significant heterogeneity was noted, and a random-effects model was utilized for combining effect sizes. The studies demonstrated that SNAGs significantly increased FRT scores, with an overall combined MD of −7.22 (95% CI: −9.38, −5.07). The MDs for short-term, medium-term, and long-term were −5.58 (95% CI: −7.97, −3.19), −9.15 (95% CI: −11.38, −6.91), and −11.00 (95% CI: −12.22, −9.78), respectively. The relationship between intervention duration and FRT scores post-SNAG and non-SNAG interventions is illustrated in Figure 5.

**Figure 6:**
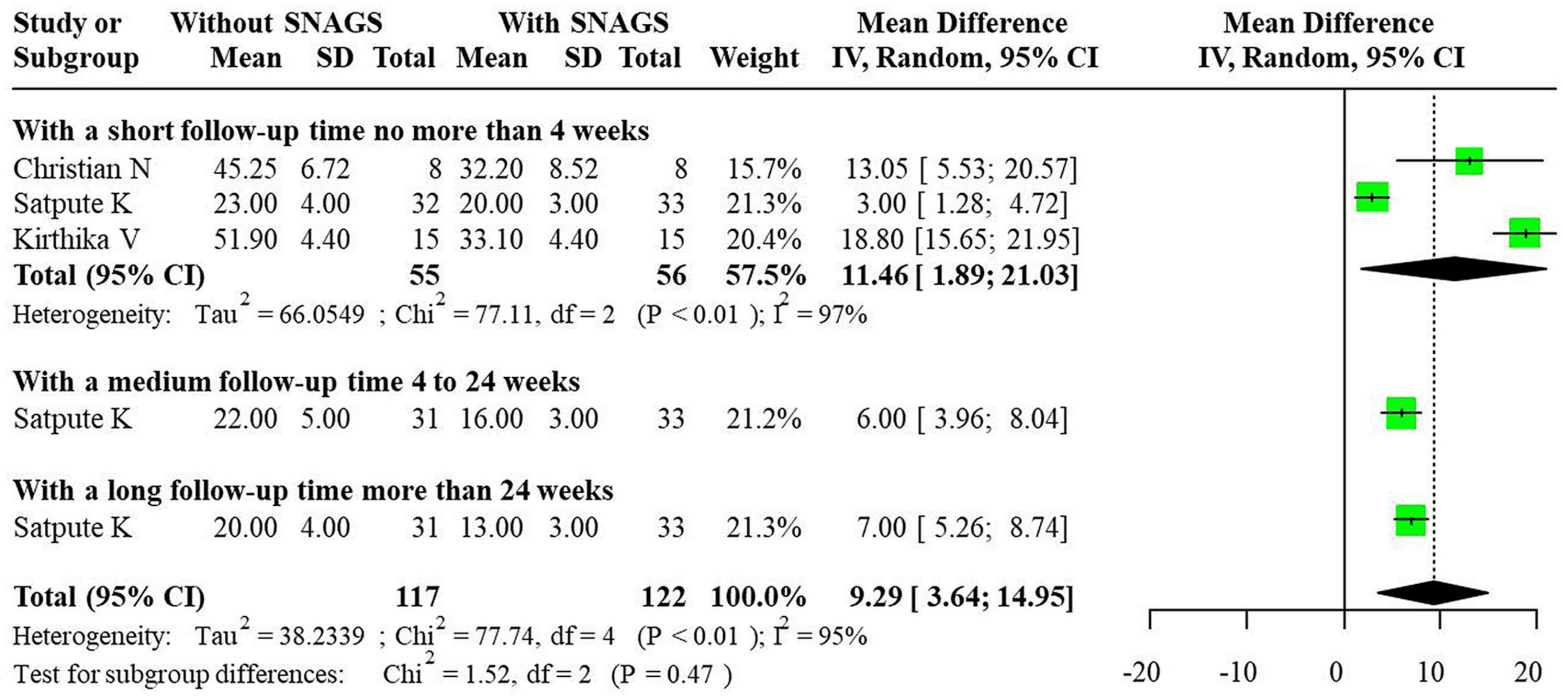
Forest plot of HDI after SNAG and non-SNAG interventions.

Regarding the HDI indicator, significant heterogeneity was observed, prompting the use of a random-effects model to combine effect sizes. The studies revealed that SNAGs significantly reduced HDI scores, with an overall combined MD of 9.29 (95% CI: 3.64, 14.95). The MDs for short-term, medium-term, and long-term were 11.46 (95% CI: 1.89, 21.03), 6.00 (95% CI: 3.96, 8.04), and 7.00 (95% CI: 3.64, 14.95), respectively. The relationship between intervention duration and HDI scores post-SNAG and non-SNAG interventions is shown in Figure 6.

**Figure 7:**
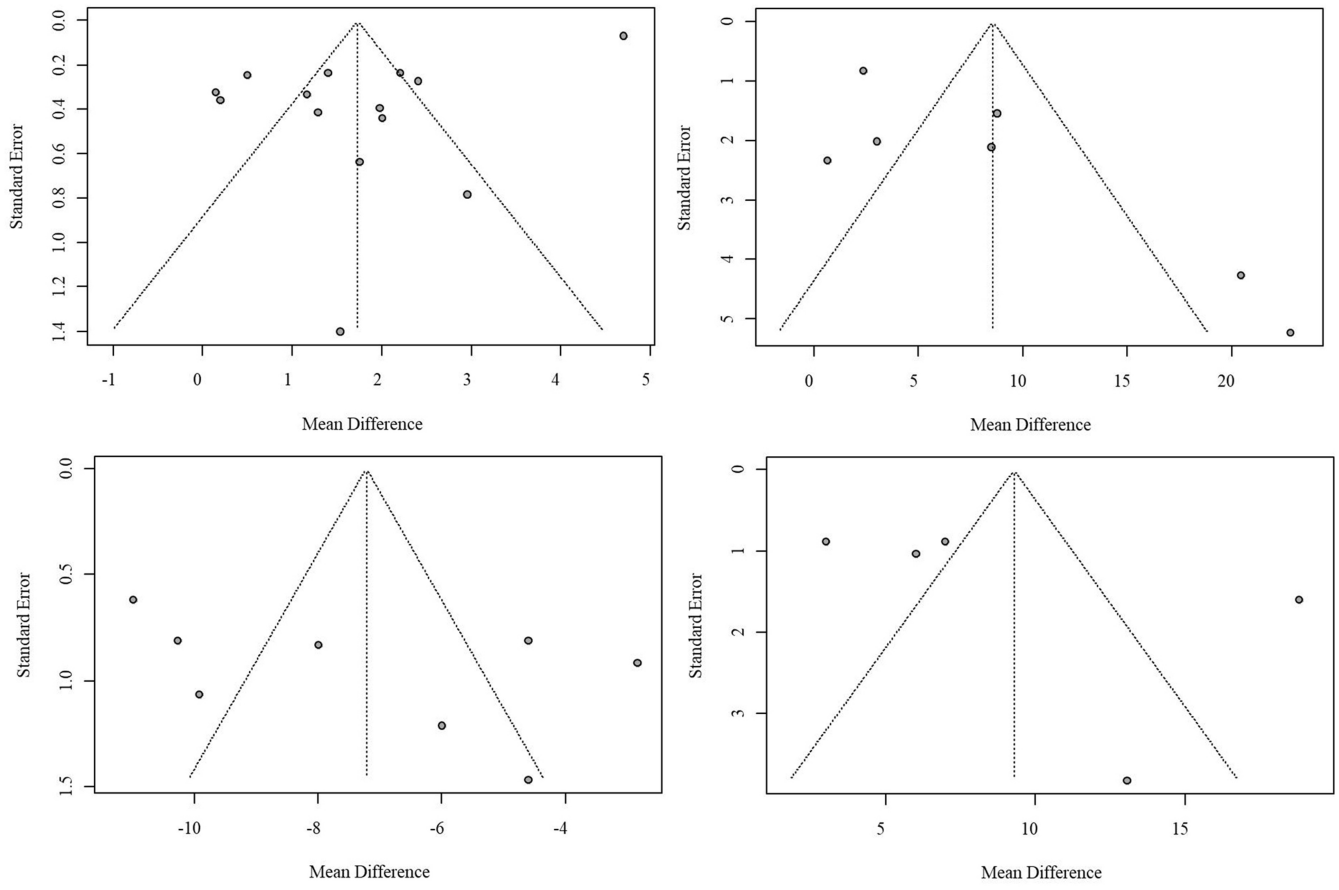
Funnel plot of publication bias in SNAG and non-SNAG indicators.

A funnel plot was constructed, and visual assessment was employed to evaluate publication bias. The funnel plot for the VAS indicator exhibited symmetry, while those for other indicators displayed some asymmetry, suggesting potential publication bias. The funnel plots for SNAG and non-SNAG indicators are presented in Figure 7.

### 3.4 Network meta-analysis results of CSM, mobilization, and massage efficacy

**Figure 8:**
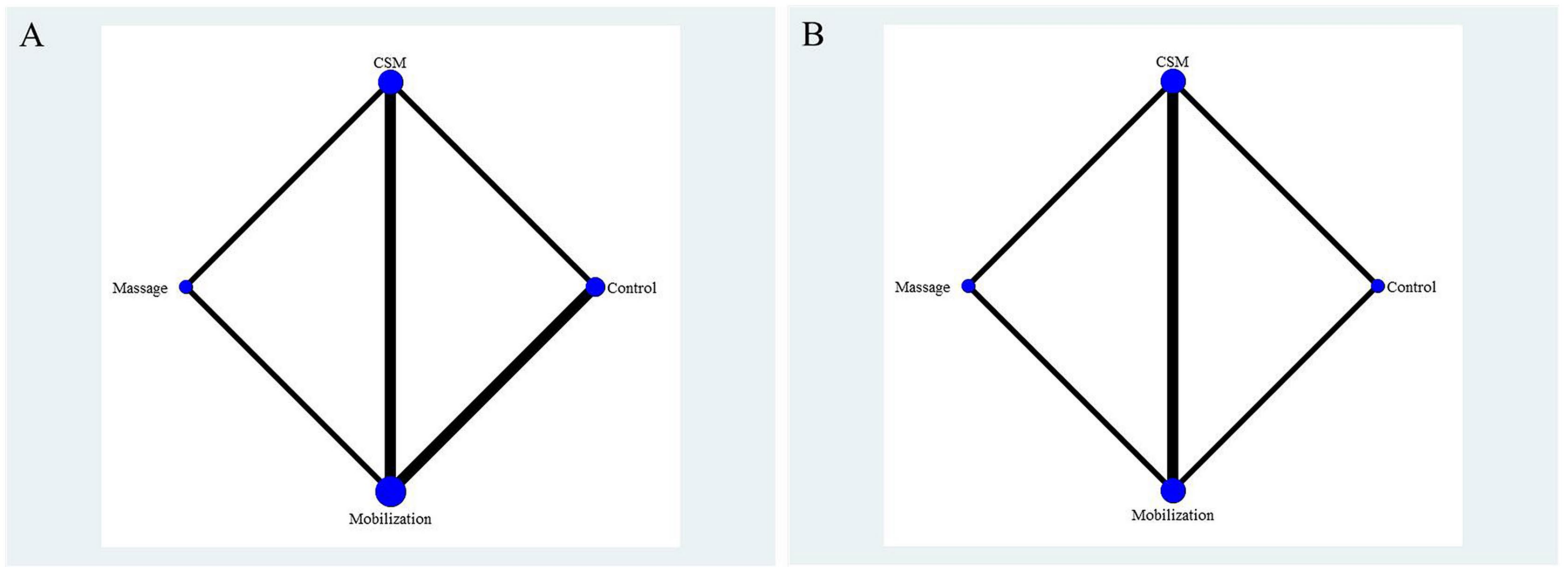
**(A)** Network plot for VAS outcomes. Direct comparisons of different interventions (CSM: cervical spine manipulation; Mobilization; Massage; Exercise) on visual analog scale (VAS) improvement. Node size represents sample size, and line thickness indicates the number of included studies. **(B)** Network plot for NDI outcomes. Direct comparisons of different interventions on Neck Disability Index (NDI) improvement. Network structure illustrates pairwise comparisons, with node and line definitions as in **(A)**.

Considering VAS and NDI as primary outcome indicators after 4 weeks of intervention, 6 articles reported enhancements in VAS following CSM, Mobilization, and Massage treatments, while 5 articles reported improvements in NDI. Direct comparisons among different intervention methods are summarized in the network relationship diagram for VAS and NDI indicators shown in Figure 8. The thickness of lines in the diagram represents the number of studies, while the size of the nodes indicates the sample size of included studies.

**Figure 9:**
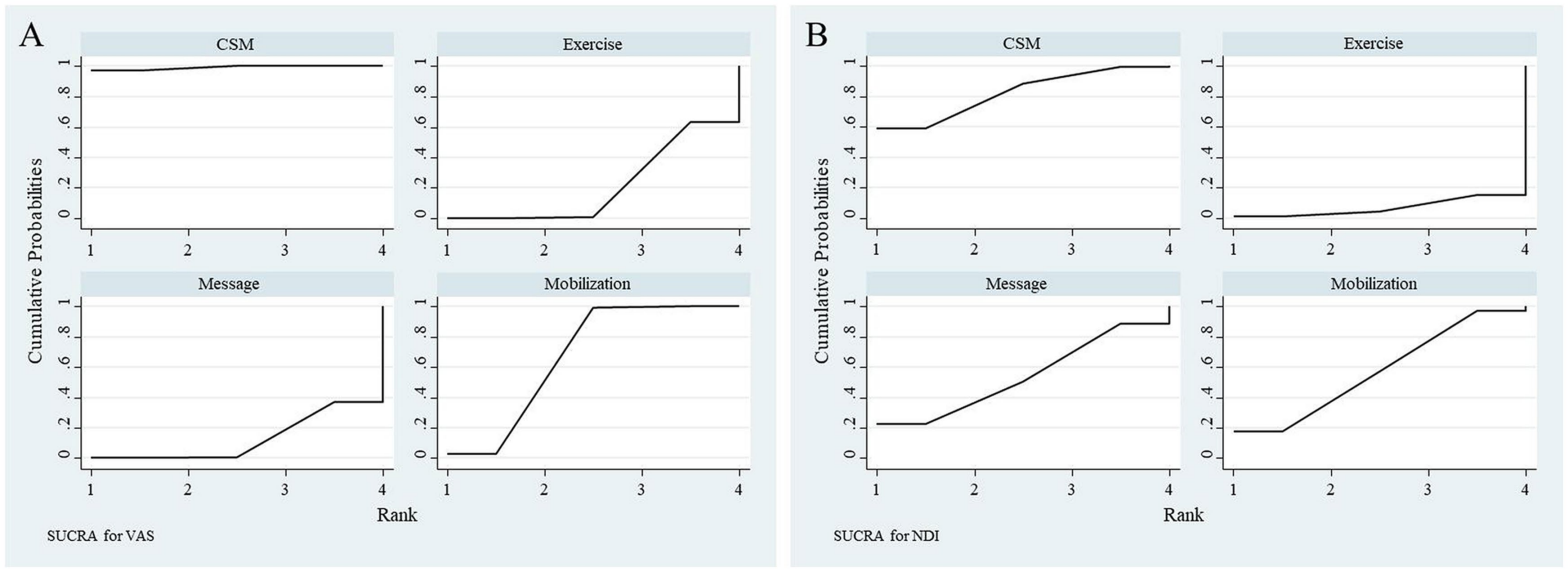
**(A)** SUCRA ranking for VAS outcomes. Surface Under the Cumulative Ranking (SUCRA) probabilities (0-100%) for interventions, showing cervical spine manipulation (CSM, 98.9%) as the most effective for VAS reduction, followed by mobilization (67.3%). **(B)** SUCRA ranking for NDI outcomes. SUCRA probabilities rank CSM (82.2%) highest for NDI improvement, with mobilization (57.2%) and massage (53.9%) as secondary options.

The SUCRA scores for various intervention methods in improving VAS scores were as follows: CSM (98.9%), Mobilization (67.3%), Exercise (21.0%), Massage (12.8%). For improving NDI scores, the SUCRA scores were: CSM (82.2%), Mobilization (57.2%), Exercise (6.7%), Massage (53.9%). CSM demonstrated superior effects in enhancing VAS and NDI. The SUCRA plots for different intervention methods for VAS and NDI indicators are presented in Figure 9.

**Figure 10:**
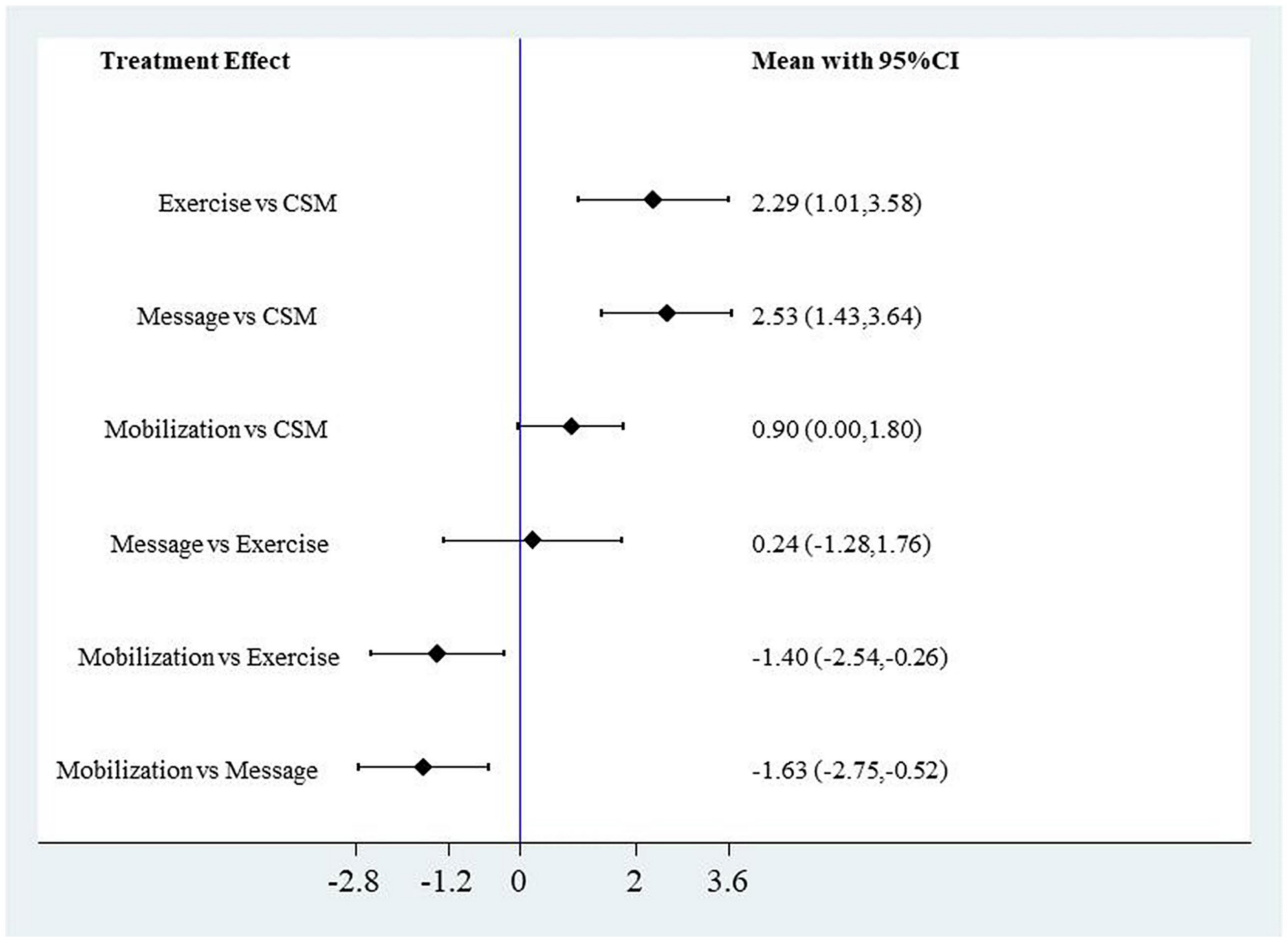
Forest plot of VAS scores for different interventions.

**Figure 11:**
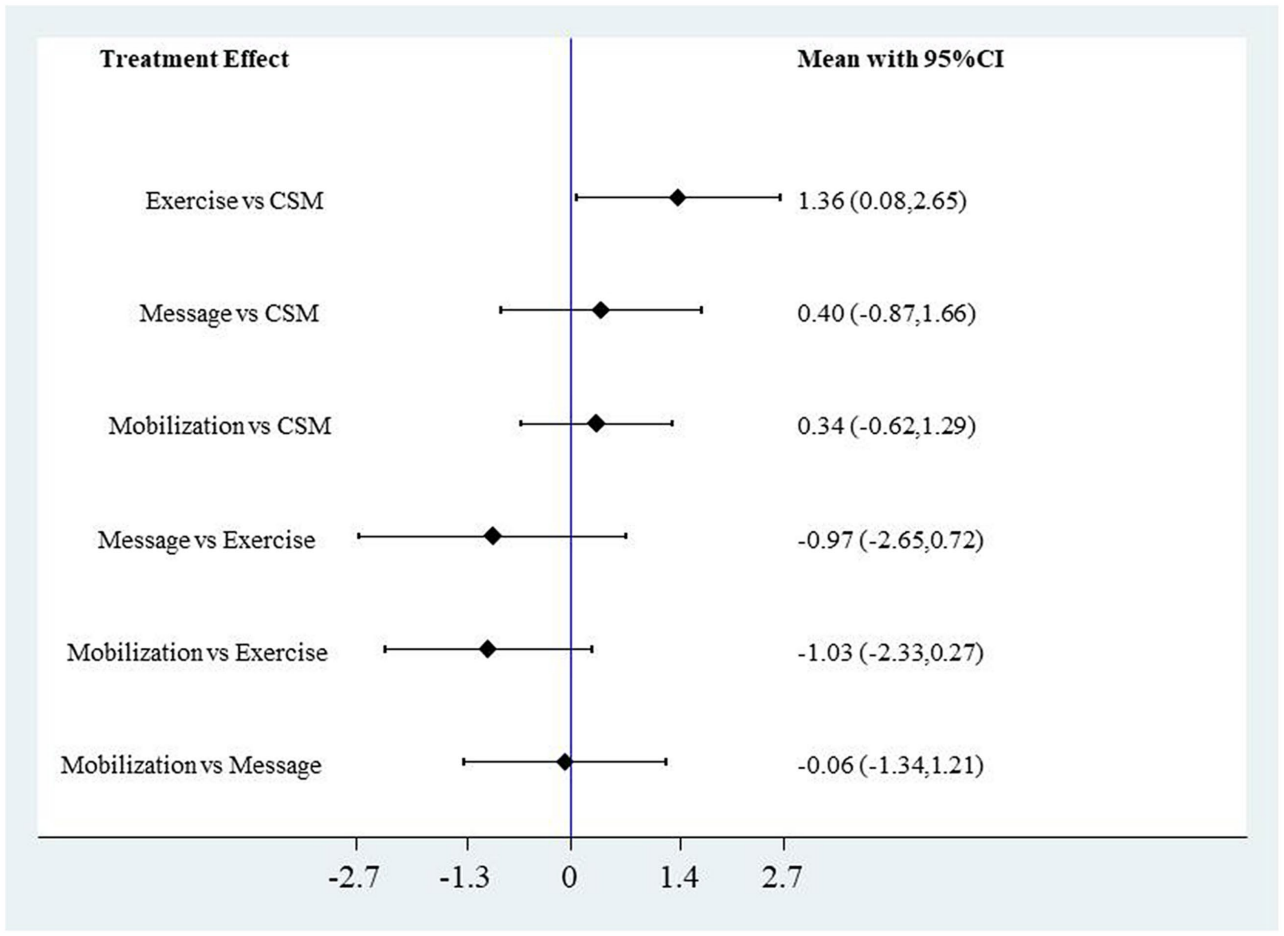
Forest plot of NDI scores for different interventions.

CSM significantly outperformed Exercise, Massage, and Mobilization in reducing VAS scores. Compared to Exercise, the MD for VAS scores was 2.29 (95% CI: 1.01, 3.58); compared to Massage, the MD for VAS scores was 2.53 (95% CI: 1.43, 3.64); compared to Mobilization, the MD for VAS scores was 0.90 (95% CI: 0.00, 1.80). Mobilization significantly outperformed Exercise and Massage in reducing VAS scores. Compared to Exercise, the MD for VAS scores was 1.40 (95% CI: 0.26, 2.54); compared to Massage, the MD for VAS scores was 1.63 (95% CI: 0.52, 2.75). The forest plot for VAS scores with different interventions is shown in Figure 10. In reducing NDI scores, CSM significantly outperformed Exercise with an MD of 1.36 (95% CI: 0.08, 2.65), while no significant differences were observed in NDI scores among the other intervention methods. The forest plot for NDI scores with different interventions is shown in Figure 11.

### 3.5 Comparing safety and effectiveness

#### 3.5.1 Effectiveness

Both cervical and thoracic spinal manipulation, as well as SNAGs, demonstrated strong efficacy in reducing pain, improving cervical range of motion, and decreasing headache frequency. Mobilization, particularly when combined with other therapies such as exercise, also yielded significant positive results. However, spinal manipulation (both cervical and thoracic) tended to provide more pronounced and faster benefits in terms of pain relief and disability reduction.

#### 3.5.2 Safety

Mobilization and SNAGs were among the safest interventions for CGH, with minimal risk of adverse effects. These methods were often preferred for patients who might not tolerate more aggressive manipulation. In contrast, spinal manipulation, particularly in the upper cervical spine, carried slightly higher risks, though these remained generally low and manageable when performed by experienced clinicians.

For patients with CGH, spinal manipulation (cervical and thoracic) and SNAGs proved to be the most effective interventions for pain relief and functional improvement. Mobilization and massage were also effective, especially when integrated with other therapies, but spinal manipulation often delivered faster and more substantial benefits. In terms of safety, mobilization and SNAGs were generally the safer options, with fewer adverse effects, while manipulation remained a highly effective choice when administered by trained professionals (Table 2).

**Table 2:** Effectiveness of manipulative interventions for CGH.

Effectiveness of manipulative interventions for CGH	Study	Findings	Safety concerns
Upper Cervical and Thoracic Manipulation	Dunning JR (25)	The study compared upper cervical and upper thoracic manipulation to mobilization and exercise for CGH. The results showed that manipulation (especially in the cervical and thoracic spine) provided significant improvements in headache frequency and severity compared to mobilization and exercise. This suggests that manipulation might be more effective in reducing CGH symptoms.	Cervical spine manipulation, particularly in the upper segments (C1-C2), can carry a small risk of adverse events, such as vertebral artery dissection or cervical artery damage. However, well-trained practitioners can minimize these risks. The studies generally report low incidence of adverse effects when these techniques are administered by experienced clinicians.
Sustained Natural Apophyseal Glides (SNAGs)	Kashif M (26), Shin EJ (33), and several others (21, 27–39)	SNAGs, specifically targeting the upper cervical spine (C1-C2), have been shown to significantly improve both headache intensity and cervical function. The studies demonstrated that SNAGs were effective in reducing the duration of headaches and improving cervical range of motion. SNAGs are particularly beneficial for those with CGH related to restricted cervical mobility.	Mobilization techniques, including SNAGs, are typically associated with fewer risks compared to high-velocity manipulation. These techniques focus on joint mobilization and range of motion rather than high-velocity thrusts. They are generally considered safe and have a low incidence of adverse effects. However, care should still be taken to ensure the appropriate application, particularly for patients with conditions like cervical instability or acute inflammation. Massage therapy is generally considered safe and carries a low risk of adverse effects, particularly when compared to more invasive manual therapies like spinal manipulation. It can be used as an adjunct to other treatments to improve muscle relaxation and alleviate tension in CGH patients.
Mobilization vs. Massage Therapy	Youssef EF (29)	The study comparing mobilization and massage therapy found that mobilization was more effective at reducing headache frequency and improving function compared to massage. While massage provided some benefit, mobilization was superior in improving cervical mobility and reducing pain intensity.	
Manipulation vs. Mobilization and Exercise	Lerner-Lentz A (21)	This study found that manipulation combined with exercise provided better outcomes in reducing both pain and disability compared to mobilization alone. It highlights the benefit of integrating manipulation with exercise to improve long-term outcomes for CGH.	
Thoracic Spine Manipulation	McDevitt AW (30)	Thoracic spine thrust manipulation was found to be effective in alleviating CGH symptoms. The study showed that thoracic manipulation improved headache frequency and severity, supporting the broader role of spinal manipulation techniques beyond just the cervical spine for CGH management.	Thoracic manipulation is considered safer than cervical manipulation in terms of risk to the cervical arteries, and adverse events are rarer. It may also be beneficial for patients who have difficulty tolerating cervical manipulation.
Cervical vs. Thoracic Spinal-Thrust Manipulation	Nambi G (31)	This study compared cervical and thoracic spinal thrust manipulation for CGH. It found that both interventions were effective, but cervical manipulation was slightly more effective in reducing headache intensity and improving cervical function. However, thoracic manipulation still showed significant benefits, especially for patients with less cervical mobility.	
Mulligan Techniques	Satpute K (32), Kirthika V (34), Khalil M (35)	Mulligan manual therapy techniques, including SNAGs, combined with exercise, significantly reduced headache frequency and intensity. Studies highlighted that Mulligan’s techniques, particularly in combination with exercise, could provide more substantial improvements compared to exercise alone, especially for reducing disability associated with CGH.	

## 4 Discussion

The current investigation represents the first direct comparison of the effects of SNAGS versus non-SNAG interventions on pain, function, and range of motion outcomes in patients with CGH. The results revealed that SNAG mobilization produced statistically significant superior improvements relative to control interventions, including reductions in VAS scores (MD = 1.73, 95%CI: 1.05, 2.40), NDI scores (MD = 8.55, 95%CI: 2.73, 14.37), FRT values (MD = −7.22, 95%CI: −9.38, −5.07), and HDI scores (MD = 9.29, 95%CI: 3.64, 14.95). Notably, these beneficial effects were maintained over time. The heterogeneity in the studies—stemming from variations in manual therapy techniques, treatment protocols (such as frequency and duration), and patient populations—can impact both the reliability and generalizability of the findings. Different therapies and treatment regimens may yield inconsistent results, making it difficult to determine which specific intervention is most effective for CGH. Additionally, the findings from one study may not be applicable to other settings or populations due to differences in patient demographics and the focus of each study. To address this heterogeneity, the article implemented several strategies: standardizing data extraction and outcome measures (such as NDI, VAS, and ROM), assessing bias risk using tools like the Cochrane Risk of Bias Tool, applying the PICOS framework to ensure comparable study features, acknowledging variability in treatment protocols, and possibly using network meta-analysis to synthesize results from different interventions. These measures help improve the consistency and applicability of the findings, despite the differences across studies.

The observed advantages of SNAG mobilization are consistent with Mulligan’s theoretical principles of manual therapy. The SNAG technique involves the application of graded mobilization along the treatment plane of the selected cervical facet joint, from the mid-range to the end-range, with the joint position maintained. This approach is theorized to reduce cervical functional impairment and improve disability by preserving the natural arthrokinematics of the zygapophyseal joints. Additionally, joint mobilization may help decrease adhesions and increase the pain pressure threshold of the paraspinal musculature. The positive effects of SNAG are likely mediated by a reduction in trigeminal cervical nucleus hyperresponsiveness and blockade of A-β fiber stimulation, which can attenuate pain and improve function (15).

The current findings align with a recent meta-analysis of 8 studies (357 participants) demonstrating that SNAG mobilization significantly improved pain, flexion-rotation, and functional outcomes in CGH patients (16). The analgesic and functional benefits of SNAG may be attributed to the stimulation of inhibitory pathways in the spinal cord and activation of descending inhibitory mechanisms in the lateral gray area around the midbrain aqueduct (17, 18).

The network meta-analysis component of this study compared the relative effects of cervical spine manipulation (CSM), mobilization, massage, and exercise on 4-week VAS and NDI outcomes. The rankwise SUCRA scores indicated that CSM had the best effects on improving both VAS (98.9%) and NDI (82.2%), suggesting it may be the most effective intervention for reducing pain and disability in CGH. CSM demonstrated significantly greater reductions in VAS compared to exercise, massage, and mobilization, while mobilization was superior to exercise and massage. For NDI, CSM was significantly better than exercise, but there were no other between-group differences.

CSM, mobilization, and massage represent common manual therapy approaches for CGH, with massage and exercise being established standard treatments (19). The rationale for CSM is that the high-velocity, low-amplitude thrust can significantly improve spinal posture and function by applying corrective forces to dysfunctional joint segments, thereby relieving pain and catalyzing the body’s natural healing processes (20). However, the literature lacks clear guidance on the optimal choice between CSM and mobilization. Interestingly, when clinician decision-making determines the interventions, the effects of CSM and mobilization appear similar, suggesting the experience and skill level of the provider may be a key determinant of outcomes (21, 22).

Several limitations warrant consideration. Both components of this study exhibited substantial heterogeneity, with variability in the frequency, duration, and nature of spinal interventions across studies. This lack of standardization complicates the translation of findings to clinical practice. Additionally, while the network meta-analysis allowed for comparative evaluation of several manual therapy modalities, the large differences between sham/control groups precluded the inclusion of SNAG, thereby limiting the comprehensiveness of the analysis.

The skill level of clinical experts can be quantified through several measures: the number of years a clinician has been practicing or the number of similar cases they have treated can give an indication of their expertise. Experience could be categorized into levels (e.g., novice, intermediate, advanced) and factored into research analysis. Certification and Training: clinicians who have additional certifications or specialized training (e.g., manual therapy certifications, advanced physiotherapy training) could be considered more skilled. Researchers could document and assess the types of training and professional development the clinicians have undergone. Standardized Skill Assessment: tools like the clinical competency examination or performance-based assessments can objectively assess the clinician’s manual therapy skills. These could include assessments of technique precision, patient interaction, and the application of evidence-based practices. Patient outcomes: an indirect measure of skill could be the clinician’s track record in improving patient outcomes, such as pain reduction, mobility improvement, or patient satisfaction. These could be tracked using outcome measures like the NDI or VAS.

To enhance the validity and reliability of future research, several methodological improvements should be considered regarding clinician training, standardization, and skill assessment. Clinician training and standardization: future studies should ensure that all clinicians involved in the study meet a standardized level of competence. This could be achieved by requiring participants to have a minimum number of hours in specific training programs or certifications related to the intervention being tested. Clinician matching across groups: to control for skill variability, studies could match clinicians across the experimental and control groups. For example, clinicians with similar levels of experience and expertise should treat both groups to minimize the impact of skill differences on treatment outcomes. Incorporating clinician skill as a variable: researchers should explicitly assess and report clinician skill level as a variable in their studies. This could involve documenting qualifications, years of experience, and certification levels, and then analyzing how these factors might influence treatment outcomes. Blinding and external review: to ensure consistency, independent external experts could review the treatment techniques and outcomes. This could provide an objective evaluation of whether treatment techniques are being applied as intended and if the clinician’s skill is impacting the results. Randomized assignment of clinicians: by randomly assigning patients to clinicians with different levels of expertise, researchers can analyze how variations in clinician skill affect treatment outcomes. This could also help in identifying the most effective clinical practices for specific techniques.

Studies on the correlation between X-ray findings and head and neck symptoms have important implications for the clinical use of manual therapy, especially in treating conditions like CGH (23, 24). X-ray imaging often reveals structural abnormalities, such as spinal misalignments, degenerative changes, or facet joint dysfunction, which may correlate with symptoms like pain, stiffness, and headaches. If a strong correlation is found between X-ray abnormalities (e.g., disc degeneration or misalignments) and symptoms, clinicians may place more focus on these factors when selecting manual therapy techniques. X-ray results can help clinicians tailor treatment plans based on structural findings. For example, if issues like joint dysfunction or facet joint problems are identified, clinicians may prioritize joint mobilizations or spinal manipulation over techniques like massage. In cases where postural issues are evident, manual therapy combined with posture correction exercises may be emphasized. When X-ray findings show a correlation with persistent or severe symptoms, it suggests that manual therapies such as spinal manipulation or mobilization could be more effective for these patients, potentially improving pain relief and functionality. Conversely, when there’s minimal correlation between X-ray findings and symptoms, soft tissue techniques like massage or muscle energy techniques may be just as beneficial, highlighting the importance of a comprehensive approach to manual therapy. X-ray imaging also helps in identifying contraindications or red flags, such as fractures or tumors, which may require modifications to treatment plans. For example, patients with severe degenerative changes might benefit more from gentle mobilizations rather than high-velocity manipulations, reducing the risk of harm. The integration of X-ray results into the overall diagnostic process can enhance treatment planning. It underscores that manual therapy should not be based solely on symptoms like pain, but should also consider underlying structural contributors. Additionally, X-rays can serve as a monitoring tool, helping clinicians track the progression of structural changes in patients with chronic conditions like CGH. This allows for adjustments in treatment to address any new complications or degeneration over time. In some cases, when X-rays indicate that structural abnormalities do not correlate with symptoms, clinicians may focus on alternative therapies such as exercise, posture correction, or ergonomic adjustments, with manual therapy being used more as an adjunct. Here, the emphasis shifts toward preventive measures to address lifestyle factors that may contribute to poor posture or movement patterns.

The small sample sizes and potential selection biases in these studies significantly limit the ability to generalize their findings to the broader CGH patient population. While the studies provide important insights into the effectiveness of manual therapy interventions, their conclusions should be interpreted cautiously. Larger, more diverse studies with more robust sampling strategies would help improve the external validity and reliability of the findings, allowing for more confident recommendations that can be applied to the wider CGH population in clinical settings.

## 5 Conclusion

In conclusion, this investigation provides evidence that SNAG mobilization can significantly improve pain, function, and range of motion in patients with CGH, with durable benefits over time. Moreover, the network meta-analysis suggests that CSM may be the most effective short-term intervention for reducing pain and disability in this population, although clinician expertise appears to be an important factor. Future research is needed to establish optimal dosing and standardization of spinal manual therapies for CGH.

## Data Availability

The original contributions presented in the study are included in the article/supplementary material, further inquiries can be directed to the corresponding author.
